# BINDER: computationally inferring a gene regulatory network for *Mycobacterium abscessus*

**DOI:** 10.1186/s12859-019-3042-8

**Published:** 2019-09-10

**Authors:** Patrick M. Staunton, Aleksandra A. Miranda-CasoLuengo, Brendan J. Loftus, Isobel Claire Gormley

**Affiliations:** 1 School of Medicine, Conway Institute, University College Dublin, Dublin, Ireland; 2 Moyne Institute of Preventive Medicine, Department of Microbiology, Trnity College Dublin, Dublin, Ireland; 3 School of Mathematics and Statistics, Insight Centre for Data Analytics, University College Dublin, Dublin, Ireland

**Keywords:** Gene regulatory network, Mycobacterium abscessus, Bayesian inference, Data integration

## Abstract

**Background:**

Although many of the genic features in *Mycobacterium abscessus* have been fully validated, a comprehensive understanding of the regulatory elements remains lacking. Moreover, there is little understanding of how the organism regulates its transcriptomic profile, enabling cells to survive in hostile environments. Here, to computationally infer the gene regulatory network for *Mycobacterium abscessus* we propose a novel statistical computational modelling approach: BayesIan gene regulatory Networks inferreD via gene coExpression and compaRative genomics (BINDER). In tandem with derived experimental coexpression data, the property of genomic conservation is exploited to probabilistically infer a gene regulatory network in *Mycobacterium abscessus*.Inference on regulatory interactions is conducted by combining ‘primary’ and ‘auxiliary’ data strata. The data forming the primary and auxiliary strata are derived from RNA-seq experiments and sequence information in the primary organism *Mycobacterium abscessus* as well as ChIP-seq data extracted from a related proxy organism *Mycobacterium tuberculosis*. The primary and auxiliary data are combined in a hierarchical Bayesian framework, informing the apposite bivariate likelihood function and prior distributions respectively. The inferred relationships provide insight to regulon groupings in *Mycobacterium abscessus*.

**Results:**

We implement BINDER on data relating to a collection of 167,280 regulator-target pairs resulting in the identification of 54 regulator-target pairs, across 5 transcription factors, for which there is strong probability of regulatory interaction.

**Conclusions:**

The inferred regulatory interactions provide insight to, and a valuable resource for further studies of, transcriptional control in *Mycobacterium abscessus*, and in the family of *Mycobacteriaceae* more generally. Further, the developed BINDER framework has broad applicability, useable in settings where computational inference of a gene regulatory network requires integration of data sources derived from both the primary organism of interest and from related proxy organisms.

## Background

*Mycobacterium abscessus* is a rapidly growing mycobacteria capable of causing a variety of soft tissue infections, primarily affecting subjects with immuno-deficiencies. *Mycobacterium abscessus* (*M. abscessus*) is considered a major pathogen involved in broncho-pulmonary infection in patients with cystic fibrosis or chronic pulmonary disease [1]. In addition, *M. abscessus* is responsible for several skin and soft tissue diseases, central nervous system infections, bacteremia, and ocular and other infections [2]. Owing to a range of cellular mechanisms, one of the most salient aspects of pathogenesis resulting from *M. abscessus* infection is its multi-drug resistance. It is the most chemotherapy-resistant rapid-growing mycobacterium [3].

While many genic features in *M. abscessus* have been fully validated and characterised in terms of the expression landscape at the transcriptional, post-transcriptional and translational levels [4], a comprehensive understanding of regulatory elements is lacking. Without functional identification of the modes of regulation present, a complete understanding of how *M. abscessus* modulates its transcriptomic tendencies, enabling cells to survive and thrive in hostile environments such as in the presence of antibiotics or in the host sputum, remains out of reach.

Gene regulatory network (GRN) resources are typically split into two categories: generalist resources and specialist resources. The former category provides regulatory information (such as transcription factors, putative and confirmed target genes/operon structures, transcription factor binding sites (TFBS) motifs, upstream location coordinates) for a wide group of organisms. CollecTF [5] is one such resource that hosts a large collection of DNA binding sites for prokaryotic transcription factors. Although CollecTF comprises a small amount of regulatory information pertaining to mycobacteria, it currently does not contain any information on *M. abscessus*. Indeed most generalist resources tend not to comprise much content on regulatory information directly relevant to *M. abscessus*.

Specialist resources tend to provide regulatory information for a much narrower subgroup of organisms such as a single species or genus; RegulonDB [6] is one such resource which comprises information regarding transcriptional regulation in *Escherichia coli*. Most resources of both types provide curation based on techniques such as SELEX-based methods [7] as well as ChIP-seq [8]. Currently, for *M. abscessus*, there is no such existing specialist resource.

Many approaches have been designed for *in silico* inference of prokaryotic GRNs. Two popular strategies for regulon mapping include (1) the use of conservation data arising from comparative genomics analyses and (2) expression data in the form of transcriptional abundance comparison. The conservation approach relies on the observation that TFBSs are often conserved between related species. This implies that regulatory resources from a given organism can be leveraged to elucidate on transcriptional control in closely related organisms [9]. Further, if two organisms with a non-distant common ancestor share an orthologous gene that is understood to assist in achieving a certain biological process (such as transcriptional regulation) in one organism, it is likely to perform a similar role in the other organism [10]. Phylogenetic footprinting provides a conservation-based approach for determining conserved noncoding sequences and associated TFBSs; such methods typically involve quantifying the rate of occurrence of noncoding DNA sequences in the upstream regions of orthologs of genes of interest in related species [11, 12].

Expression-based approaches tend to model the expression of a target gene candidate as a function of the expression or activation of a regulator gene. The GENIE3 [13] method frames the problem of deriving a regulatory network between *p* genes as *p* different regression tree-based ensemble models where the expression pattern of one gene is predicted by the expression pattern of all other genes in the collection. Other authors have noted the observed property that genes sharing a common network have a greater tendency to exhibit strong coexpression [14]. Weighted correlation network analysis (WGCNA) [15] is a software package that implements a suite of correlation-based methods for describing the coexpression patterns among genes across experimental samples designed with a view to uncovering gene networks of several varieties.

The literature on prokaryotic gene regulation is replete with ChIP-seq experiments detailing the specifics of transcriptomic control [16, 17]. ChIP-seq provides a means of isolating target DNA sequences and transcription factor bound protein complexes stimulated in response to induced transcription factor production. This process facilitates the ascertaining of relationships between specific transcription factors and target binding site DNA sequences (including their downstream genic and intergenic units). Such data are not presently available for *M. abscessus*, due to its status as an emerging pathogen [3]. However, similar resources exist to varying degrees of completeness for closely related organisms, such as those in the family of Mycobacteriaceae [18, 19]. Many efforts have focussed on the integration of ChIP-seq experimental data with RNA-based expression results to improve GRN inference [20].

In general, the concept of designing hybrid models that integrate existing regulatory information and expression abundance results has been the focus of much research. For example, iRafNet [21] implements a random forest approach to inferring GRNs while incorporating prior regulatory knowledge such that putative regulators used to build individual trees are sampled in accordance with the provided prior information. GRACE [22] integrates biological a priori data as well as heterogeneous data and makes use of Markov random fields to infer regulatory networks in eurkaryotic organisms. The RNEA [23] approach also combines prior knowledge from manual literature curation and experimental data with enrichment analysis to infer relevant subnetworks under experimental conditions. The multi-species cMonkey approach [24] includes gene expression data for multiple related organisms in addition to upstream sequence information and other network knowledge, iteratively building biclusters to detect putative co-regulated gene groupings.

Hierarchical Bayesian frameworks provide a natural choice for heterogenous data integration; Bayesian methods like COGRIM [25] and CRNET [26] have sought to exploit this quality. With a view to inferring GRNs, integrative Bayesian methods have focussed on directly modelling putative target gene expression data as a function of regulator activity in addition to binding strength and sequence information.

Herein, we introduce a novel statistical modelling approach to computationally inferring the GRN for *M. abscessus*: BayesIan gene regulatory Networks inferreD via gene coExpression and compaRative genomics (BINDER). BINDER is an integrative approach, hybridising coexpression data and comparative genomics profiles to infer prokaryotic regulons. BINDER requires two organisms: an organism of interest, here *M. abscessus*, and an annotated proxy organism, here *Mycobacterium tuberculosis* (*M. tuberculosis*). To computationally infer the GRN for *M. abscessus* we leverage existing resources: specifically we exploit several RNA-seq libraries elicited from *M. abscessus* generated across a range of experimental conditions, and the unique availability of a high-quality and comprehensively catalogued ChIP-seq-derived regulatory network in *M. tuberculosis* [27]. BINDER utilises a primary data stratum and an auxiliary data stratum. Here, the data forming the primary and auxiliary strata are derived from RNA-seq experiments and sequence information from *M. abscessus* as well as ChIP-seq data extracted from the related *M. tuberculosis*. BINDER is a Bayesian hierarchical model that appositely models the type and structure of both this primary and auxiliary data to infer the probability of a regulatory interaction between a regulator-target pair. The auxiliary data inform the prior distributions and the posterior distributions are updated by accounting for the primary coexpression data in a novel, apposite bivariate likelihood function. BINDER’s Bayesian framework facilitates the borrowing of information across the genome yielding estimates of the probability of regulation between regulator and target candidate genes, as well as quantification of the inherent uncertainty in a probabilistically principled manner.

In what follows, we explore the performance of BINDER under a range of challenging simulated data settings, as well as in two case studies using *Bacillus subtilis* (*B. subtilis*) and *Escherichia coli* (*E. coli*) as the primary organisms of interest, for which regulatory interactions have been well-established. We present the regulatory interactions inferred on *M. abscessus* by BINDER, and explore in detail the putative inferred regulon corresponding to the transcriptional regulator zur. We also include an exploration of prior sensitivity concerns and some discussion. The “Methods” section describes the data utilised and details the architecture of the BINDER approach.

The results of this effort provide insight to, and a valuable resource for further studies of, transcriptional control in *M. abscessus*, and in the family of *Mycobacteriaceae* more generally. Further, the developed BINDER framework has broad applicability, useable in settings where computational inference of a GRN requires integration of data sources derived from both the primary organism of interest and from a related proxy organism. A software implementation for BINDER is provided by its associated R package, which is freely available from github.com/ptrcksn/BINDER.

## Results

### Exploring *M. abscessus* and *M. tuberculosis* shared orthology

It has been established that there is high retention of gene regulation in prokaryotes between species [28]. Moreover, it has been demonstrated that gene function is also retained across wide phylogenetic distances in prokaryotes [29]. Given the availability of a large number of experimentally validated regulatory networks in *M. tuberculosis* [27], from the standpoint of inferring a GRN in *M. abscessus* using conservation phenomena, we quantifed the extent to which genes present in *M. tuberculosis* are conserved in *M. abscessus*. To do so, we employ the Ortholuge [64] procedure which facilitates bacterial and archaeal comparative genomic analysis and large-scale ortholog predictions. Through Ortholuge, we categorise orthologs as belonging to one of five tiers, ranging from more reliable to less reliable: supporting-species-divergence (SSD), borderline supporting-species-divergence (borderline SSD), reciprocal best blast (RBB), similar non-supporting-species-divergence (similar non-SSD) and non-supporting-species-divergence (non-SSD). We found 1343 SSD putative orthologs, 116 borderline SSD putative orthologs, 845 genes that satisfied the RBB criteria but did not undergo any further analysis, 6 similar non-SSD putative orthologs and 85 non-SSD putative orthologs. In total, we found 2395 predicted orthologs of all qualities, equating to ≈ 48% of all annotated genes in *M. abscessus*.

**Fig. 1:**
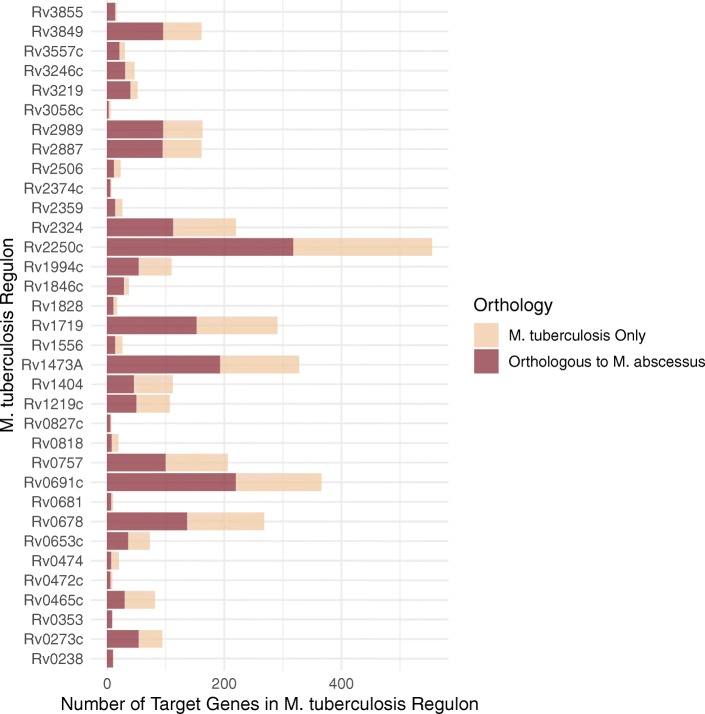
Number of target genes in the 34 orthologous *M. tuberculosis* regulons. Also illustrated is the the extent of orthology between *M. tuberculosis* and *M. abscessus*

In terms of regulatory interactions, for 34 orthologous regulators of interest and where possible, we performed a one-to-one mapping of all validated regulatory interactions in *M. tuberculosis* to their corresponding orthologs in *M. abscessus*. We found a mean regulon size in *M. tuberculosis* of 107.91 genes (sd: 128.78) (standard deviations in parentheses). Of these 34 regulons, the mean regulon proportion comprising orthologous interactions in *M. abscessus* is 0.61 (sd: 0.16) (Fig. 1). These results are suggestive of conserved regulatory interactions between *M. tuberculosis* and *M. abscessus*.

### BINDER simulation study

In order to evaluate the performance of BINDER (“The BINDER model for inferring a GRN” section), we perform a simulation study across a number of settings. Our focus is on exploring the impact of BINDER’s hierarchical Bayesian model structure and on the influence of the inclusion of the auxiliary data when inferring a GRN. Specifically we focus on the parameter *θ*_*r, t*_ representing the probability of an interaction in the (*r, t*)th regulator-target pair and consider two simplified versions of the BINDER model:

- *Deterministic model*: each *θ*_*r, t*_ is modelled deterministically as a linear function of the auxiliary data. Thus BINDER’s prior on *θ*_*r, t*_ is replaced by:
  $$\begin{array}{@{}rcl@{}} \text{logit}(\theta_{r,t}) = \zeta_{r} + \tau_{\text{ME}_{r}} \text{ME}_{r,t} + \tau_{\text{PE}_{r}} \text{PE}_{r,t} \end{array} $$
- *Non-auxiliary model*: no auxiliary data are used during inference on *θ*_*r, t*_, which are instead inferred based on the primary data only. In this case BINDER’s prior on *θ*_*r, t*_ is instead replaced by the prior $\text {logit}(\theta _{r,t}) \sim \mathcal {U}(-\infty,\infty)$.

In addition, the impact on inference of noisy primary data and of large variability in the true underlying *θ*_*r, t*_ parameters is also of interest. Since the primary data CP and CM are assumed to be $\mathcal {N}_{l}(\text {logit}\left (\theta _{r,t}),\psi _{k_{r}} \right)$ for *k*∈{CP, CM}, larger values of $\psi _{k_{r}}$ reflect noisier primary data. Similarly, $\text {logit}(\theta _{r,t}) \sim \mathcal {N}\left (\gamma _{r,t}, \phi _{r}\right)$, with larger values of *ϕ*_*r*_ reflecting larger variation in the underlying regulatory interaction probabilities. Hence, we compare the performance of BINDER, the deterministic model and the non-auxiliary model on 9 distinct dispersion parameterisations corresponding to the Cartesian product of $\phantom {\dot {i}\!} \boldsymbol {\psi _{r}} = \{\psi _{\text {CM}_{r}}, \psi _{\text {CP}_{r}}\} = \{\text {low} = 1, \text {mid} = 2, \text {high} = 3\}$ and *ϕ*_*r*_={low=1,mid=2,high=3}.

For each of the nine dispersion settings, we simulate three data sets, each with *N*=1,000 regulator-target pairs. To challenge the BINDER model, we consider weakly informative auxiliary data: ME and PE are generated from a Bernoulli distribution with success parameter 0.1. We compute *γ*_*r, t*_ according to (1) where $\left (\zeta _{r}, \tau _{\text {ME}_{r}},\tau _{\text {PE}_{r}}\right) = (-3.5, 3.8, 2.9)$ and simulate $ \text {logit}(\theta _{r,t}) \sim \mathcal {N}(\gamma _{r,t},\phi _{r})$. Finally, for the primary data, we simulate $\text {CM}_{r,t} \sim \mathcal {N}_{l}(\text {logit}\left (\theta _{r,t}),\psi _{\text {CP}_{{r}}}\right)$ and $\text {CP}_{r,t} \sim \mathcal {N}(\text {logit}(\theta _{r,t}),\psi _{\text {CM}_{{r}}})$. Model performance across the 27 settings considered was assessed using the mean absolute deviation (MAD) [30] between each true simulated *θ*_*r, t*_ and its resulting posterior mean estimate.

**Fig. 2:**
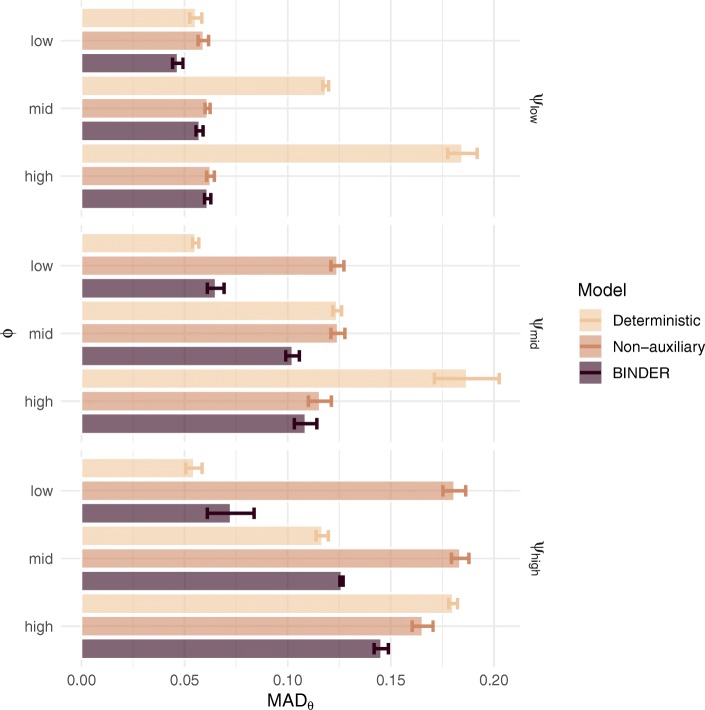
Simulation results illustrating the mean absolute deviation (MAD) between the true and estimated regulation interaction probabilities achieved by the deterministic, non-auxiliary and BINDER approaches across a range of dispersion parameter settings

We observed competitive performance of the BINDER approach over both the deterministic and non-auxiliary approaches for the majority of settings considered in terms of lower MAD (Fig. 2). Specifically, the mean for the MAD statistics for the BINDER approach was 0.087 (sd: 0.034) as compared with 0.120 (sd: 0.050) and 0.120 (sd: 0.056) for the deterministic and non-auxiliary approaches respectively. The deterministic approach has a tendency to perform worse in instances where the dispersion around each *θ*_*r, t*_ value is large (i.e. high values for *ϕ*_*r*_). This is to be expected as the deterministic approach has insufficient flexibility to model *θ*_*r, t*_ values that lie distant from their mean value resulting in higher MAD statistics. On the contrary, the deterministic approach does well in the setting of low *ϕ*_*r*_. In contrast, the non-auxiliary approach tends to be less sensitive to changes in the dispersion around the mean of the distribution of *θ*_*r, t*_. However, given that the non-auxiliary approach only uses the primary data to infer *θ*_*r, t*_, when the level of dispersion around the mean of CP and CM is high (i.e. high values for ***ψ***_***r***_) the primary data contain a weaker signal leading to poor estimation of the true *θ*_*r, t*_ and resulting in higher MAD statistics. As a compromise between the deterministic and non-auxiliary approaches, BINDER utilises the information contained in the auxiliary data whilst, simultaneously, providing the flexibility to accommodate observation-specific variation in the regulation interaction probabilities resulting in more accurate inference. BINDER outperforms the non-auxiliary model in all settings considered, and is only marginally outperformed in a minority of cases by the deterministic model in settings where *ϕ*_*r*_ is mid or low.

### Application of BINDER to *Escherichia coli* and *Bacillus subtilis* data

As a benchmarking exercise to assess the performance of BINDER on a *bona fide* regulatory interaction data set, we investigated BINDER’s ability to infer interaction plausibility for the fur and lexA regulons in *Escherichia coli* [31] and *Bacillus subtilis* [32]. Where *E. coli* constitutes the organism of interest, *Pseudomonas aeruginosa* (*P. aeruginosa*) [33] constitutes the proxy organism and where *B. subtilis* is the organism of interest, *Listeria monocytogenes* (*L. monocytogenes*) [34] fulfils the role of the proxy organism. Considering two regulons across these well-researched settings allows for intra-regulon and inter-regulon analysis as well as intra-organism and inter-organism analysis.

The ferric uptake regulator, or fur, is a transcriptional factor originally described as a repressive regulator of genes involved in iron import. Since then, aside from iron-homeostasis, fur has been shown to be associated with processes such as resistance to oxidative stress, pH homeostasis and quorum sensing as well as other cellular mechanisms [35]. In bacteria, the SOS response provides the means for responding to DNA damage; the expression of genes comprising the SOS regulatory network is under the control of lexA [36]. lexA is a global transcription factor that undergoes cleavage during stress permitting expression of DNA repair functions [37]. lexA also regulates genes that are not comprised within the SOS response program [36].

Here we avail of well-established regulator-target interactions as detailed by RegulonDB [6] for *E. coli* and well-established regulator-target interactions as per SubtiWiki [38] for *B. subtilis*. To build the primary data, we used *E. coli* expression data from COLOMBOS [39] and *B. subtilis* expression data from SubtiWiki [40]. For the auxiliary data, we use regulatory sequence motifs and orthologous target interactions from *P. aeruginosa* and *L. monocytogenes* curated by collecTF [5].

We consider the BINDER, deterministic and non-auxiliary approaches to infer the GRNs in *Escherichia coli* and in *Bacillus subtilis* from their primary and auxiliary data. Non-informative priors were employed with mean hyperparameters set to 0 and standard deviation hyperparameters set to 3, with the exception of the prior on *ϕ*_*r*_ which was set to $\phi _{r} \sim \mathcal {N}_{(0, \infty)}(1, 0.1)$ for regularisation purposes. Further, we also consider iRafNet [21] which employs an integrative prior-information-based approach to random forest inference of GRNs from expression data. For iRafNet, we applied the algorithm to each target candidate of interest individually using the fur and lexA regulator genes as predictors; further, in addition to the standardised expression matrix, for the iRafNet prior information matrix *W*, the element *w*_*ij*_, corresponding to the *i*th regulator and *j*th target candidate, was configured such that *w*_*ij*_=exp(1) if ME=1 or PE=1 and *w*_*ij*_=exp(0) for *i*≠*j*.

In total, of the 4221 uniquely labelled genes present in RegulonDB with available expression data, 67 correspond to well-established regulatory interactions concerning fur and 23 correspond to well-established interactions concerning lexA in *E. coli*. For *B. subtilis*, of the 4162 uniquely labelled genes with available expression data, 58 correspond to well-established regulatory interactions with fur and 57 to well-established regulatory interactions with lexA.

**Fig. 3:**
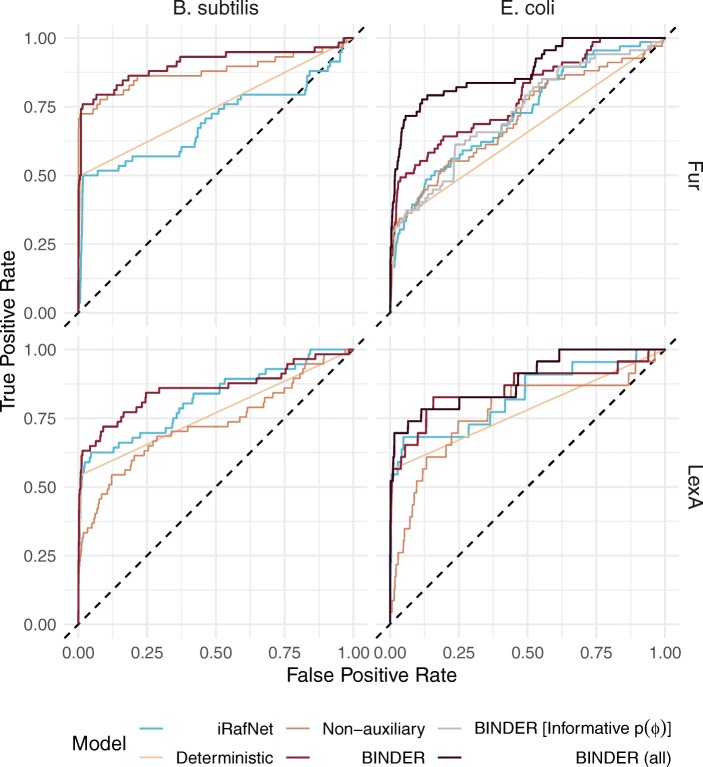
ROC analysis for $\theta _{r,t}^{50\%}$ posterior estimates for the BINDER, deterministic and non-auxiliary approaches and gene importance estimates for iRafNet for the *r*= fur and *r*= lexA regulons in *E. coli* and *B. subtilis*. BINDER (all) denotes results from analysis of BINDER applied to the complete coexpression data; BINDER relates to its application to the reduced data set

For the fur regulon in *E. coli*, BINDER achieved an area under curve (AUC) of 0.880. Notably however, in contrast to BINDER, iRafNet omits data recorded under conditions for which expression levels for all genes are not available. Thus, in order to fairly compare performance with iRafNet, we applied BINDER to a reduced expression matrix comprising fewer conditions such that no missing data were present. BINDER achieved an AUC of 0.787 as compared with 0.710, 0.654 and 0.725 for the non-auxiliary, deterministic and iRafNet approaches respectively (Fig. 3, Table 1).

**Table 1:** AUC scores achieved by each modelling approach for each regulon in each organism

Model	fur (*E. coli*)	lexA (*E. coli*)	fur (*B. subtilis*)	lexA (*B. subtilis*)
iRafNet	0.725	0.829	0.694	0.819
Deterministic	0.654	0.778	0.746	0.767
Non-auxiliary	0.710	0.768	0.878	0.728
BINDER	0.787	0.857	0.905	0.855
BINDER (all)	0.880	0.888	-	-
BINDER (informative *p*(*ϕ*))	0.729	-	-	-

**Fig. 4:**
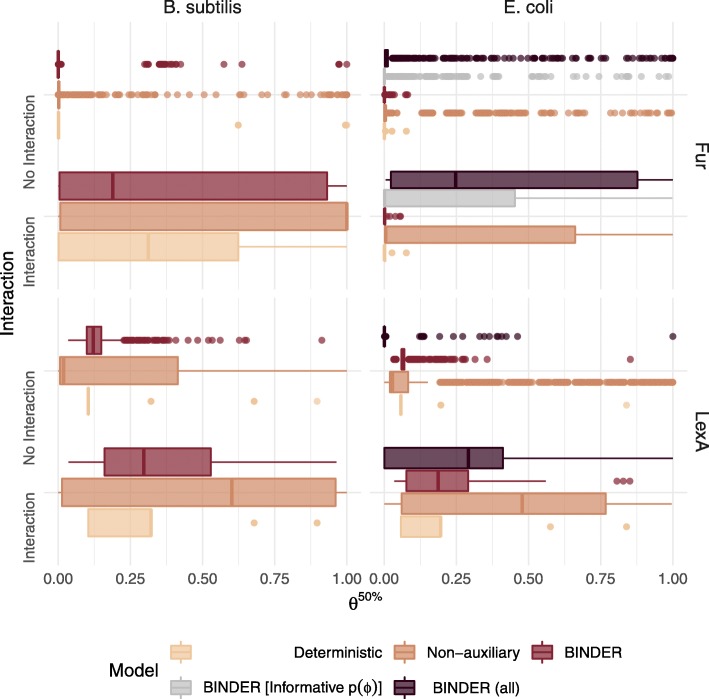
Posterior estimates of $\theta _{r,t}^{50\%}$ for the BINDER, deterministic and non-auxiliary approaches for *r*= fur and *r*= lexA regulons in *E. coli* and *B. subtilis*, factored by established interaction status

Interestingly, for BINDER applied to the reduced coexpression data, the mean posterior 50th percentile *θ*fur,*t*50*%*∀*t*∈*T* corresponding to validated regulatory interactions was only 0.0050 as compared with 0.0016 for the mean *θ*fur,*t*50*%* corresponding to observations without evidenced regulatory interactions (Fig. 4). That this BINDER implementation achieved a corresponding AUC of 0.787 suggests that the distribution of *θ*fur,*t*50*%* values is highly skewed to the right, and thus their relative magnitude is of importance when observing BINDER’s output. Interestingly, we did not observe this effect when BINDER was applied to the complete expression data. Thus, we imposed a more informative prior $\phi _{\text {fur}} \sim \mathcal {N}_{(0,\infty)}(10, 0.01)$ and applied BINDER again resulting in a mean *θ*fur,*t*50*%* corresponding to validated regulatory interactions of 0.2427 as compared with 0.0183 for the mean *θ*fur,*t*50*%* corresponding to observations without evidenced regulatory interactions (Fig. 4). However, with this informative prior the AUC dropped to 0.729. This is almost identical to the AUC for the non-auxiliary implementation which is intuitive because as *ϕ*_fur_ increases, the auxiliary stratum provides diminishing influence (Fig. 3, Table 1).

For the lexA regulon in *E. coli*, BINDER achieves an AUC of 0.888. Once again, in order to compare performance with iRafNet, we re-applied BINDER to a reduced expression matrix comprising fewer conditions such that no missing data were present. For the reduced expression data BINDER achieved an AUC of 0.857 as compared with 0.768, 0.778 and 0.829 for the non-auxiliary, deterministic and iRafNet approaches respectively (Fig. 3, Table 1).

Performance was similar for the *B. subtilis* organism (Fig. 3, Table 1). For the fur regulon, BINDER achieved an AUC of 0.905 as compared with 0.878, 0.746 and 0.694 for the non-auxiliary, deterministic and iRafNet approaches respectively. For the lexA regulon, BINDER achieves an AUC of 0.855 as compared with 0.728, 0.767 and 0.819 for the non-auxiliary, deterministic and iRafNet approaches respectively.

Not only does BINDER out perform all other considered approaches in terms of AUC, but, considering false positive rates in the neighbourhood of 0, BINDER tends to achieve higher true positive rates than any of the other approaches. This is particularly important because, owing to sparse regulatory connectivity across a given genome, regulon mapping is typically a minority class problem i.e. the vast majority of target candidates will constitute negatives for most regulators. This implies that a low false positive rate can still translate to a large *number* of false positives.

**Fig. 5:**
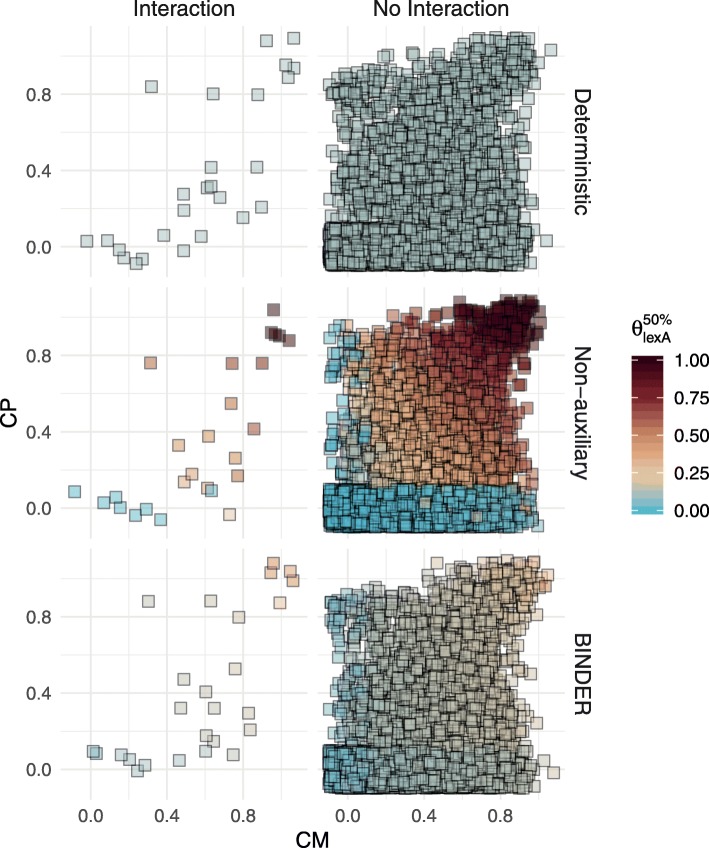
For the lexA regulon in *B. subtilis* and for targets where the auxiliary data ME=0 and PE=0, estimates of $\theta _{\text {lexA},t}^{50\%}$ for the BINDER, deterministic and non-auxiliary approaches, factored by known interaction status. The primary data values are CM and CP; points are jittered slightly for visibility

The ability of BINDER to integrate and borrow information across primary and auxiliary data when inferring a GRN is demonstrated in Fig. 5 for the particular case of the lexA regulator in *B. subtilis* when there is no auxiliary evidence. Only the full BINDER implementation is capable of tempering estimates when there is disagreement between interaction status and auxiliary evidence; when there is an interaction but no auxiliary evidence BINDER is capable of exploiting the individual primary data values, CM and CP, to provide higher estimates to the regulator-target candidate; however, the deterministic approach lacks the flexibility to provide any high $\theta _{\text {lexA},t}^{50\%}$ estimates in the absence of auxiliary evidence. Similarly, owing to the lack of auxiliary evidence, BINDER is capable of tempering its estimates for $\theta _{\text {lexA},t}^{50\%}$ when there is no interaction and no auxiliary evidence; in contrast, the non-auxiliary approach results in high $\theta _{\text {lexA},t}^{50\%}$ estimates for all observations with high primary data values CM and CP. BINDER’s hierarchical modelling structure and ability to borrow local *and* global information from both the primary and auxiliary data sources results in more realistic estimates: higher $\theta _{\text {lexA},t}^{50\%}$ estimates for putative interactions and lower $\theta _{\text {lexA},t}^{50\%}$ estimates for putative non-interactions in general. Synoptically, BINDER’s ability to integrate the information on whether a given regulator-target pair has an affinity for the predicted motif and/or an orthologous regulatory interaction in the proxy organism with the information provided in the primary data stratum provides greater flexibility.

### Application of BINDER to *M. abscessus* data

With a view to producing a model of regulation in *M. abscessus*, we leveraged data from across 34 orthologous ChIP-seq validated interactions in *M. tuberculosis* and from 32 RNA-seq libraries from across 16 distinct experimental conditions in *M. abscessus*. We considered *R*=34 orthologous regulators in *M. tuberculosis*, and *T*=4920 target candidates in the *M. abscessus* genome, yielding *N*=167,280 regulator-target pairs. For computational efficiency, given the likelihood function can be factored by regulator, we run BINDER on the *R*=34 orthologous regulators’ data in parallel. To computationally infer the gene regulatory network for *M. abscessus* the posterior distribution *p*(*θ*_*r, t*_|…) is of key interest, for *r*∈*R* and *t*∈*T* with … denoting all auxiliary and primary data and other model parameters.

#### Prior sensitivity analysis

In order to assess the sensitivity of inference to the prior distribution specifications, we constructed three different prior parameterisation settings and compared the resulting inferences. The three settings considered were labelled as ‘non-informative’, ‘informative’ and ‘precise’ (Table 2). In particular, the informative settings reflect a priori beliefs that: (1) the auxiliary data PE and ME would encode a reliable positive indication as to whether a given regulatory interaction exists and (2) a negative intercept would be required to correctly model interaction plausibility. The precise setting reflects more extreme versions of the informative setting (in terms of smaller auxiliary data scale hyperparameters).

**Table 2:** Prior parameterisation settings considered for sensitivity analysis of BINDER

Hyperparameter	Uninformative	Informative	Precise
$\mu _{\zeta _{r}}$	0	-3	-3
$\sigma _{\zeta _{r}}$	3	1	0.1
$\mu _{\tau _{\text {ME}_{r}}}$	0	3	3
$\sigma _{\tau _{\text {ME}_{r}}}$	3	1	0.1
$\mu _{\tau _{\text {PE}_{r}}}$	0	3	3
$\sigma _{\tau _{\text {PE}_{r}}}$	3	1	0.1
$\mu _{\phi _{r}}$	0	0	0
$\sigma _{\phi _{r}}$	1	0.5	0.1
$\mu _{\psi _{\text {CP}_{r}}}$	0	0	0
$\sigma _{\psi _{\text {CP}_{r}}}$	3	1.5	0.5
$\mu _{\psi _{\text {CM}_{r}}}$	0	0	0
$\sigma _{\psi _{\text {CM}_{r}}}$	3	1.5	0.5

**Fig. 6:**
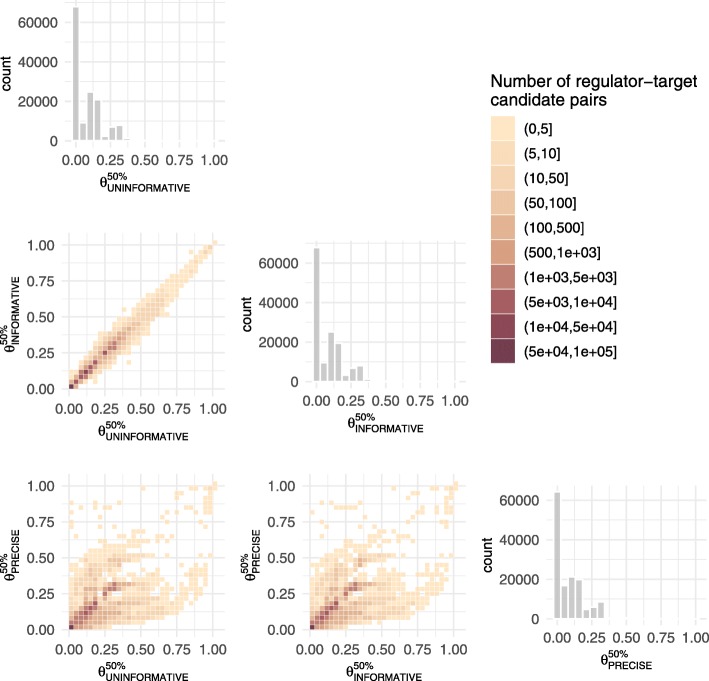
Heat map illustrating the similarity between mean predicted $\theta _{r,t}^{50\%}$ values achieved by BINDER under three distinct prior distribution parameterisations (uninformative, informative, precise) on the set of *N*=167,280 regulator-target pairs

Inference was relatively insensitive to prior specification in terms of MAD scores for $\theta _{r,t}^{50\%}$ (uninformative versus informative: 0.0040, sd: 0.0094; uninformative versus precise: 0.0183, sd: 0.0466; informative versus precise: 0.0168, sd: 0.0437, Fig. 6). Using a classification criterion such that regulator-target pairs with a posterior 50th percentile $\theta _{r,t}^{50\%} > 0.9$ are classified as positive regulation cases, comparing uninformative to informative positive regulation cases yielded an adjusted Rand index [41] of 0.9247, versus 0.5203 and 0.5553 for uninformative versus precise and informative versus precise respectively (an adjusted Rand index of 1 indicates perfect agreement). Thus, for the remainder of this work, with a view to allowing the data to determine the parameter estimates without imposing strong beliefs, we focus on the uninformative parameterisation.

#### Inferred regulatory interactions in *M. abscessus*

**Fig. 7:**
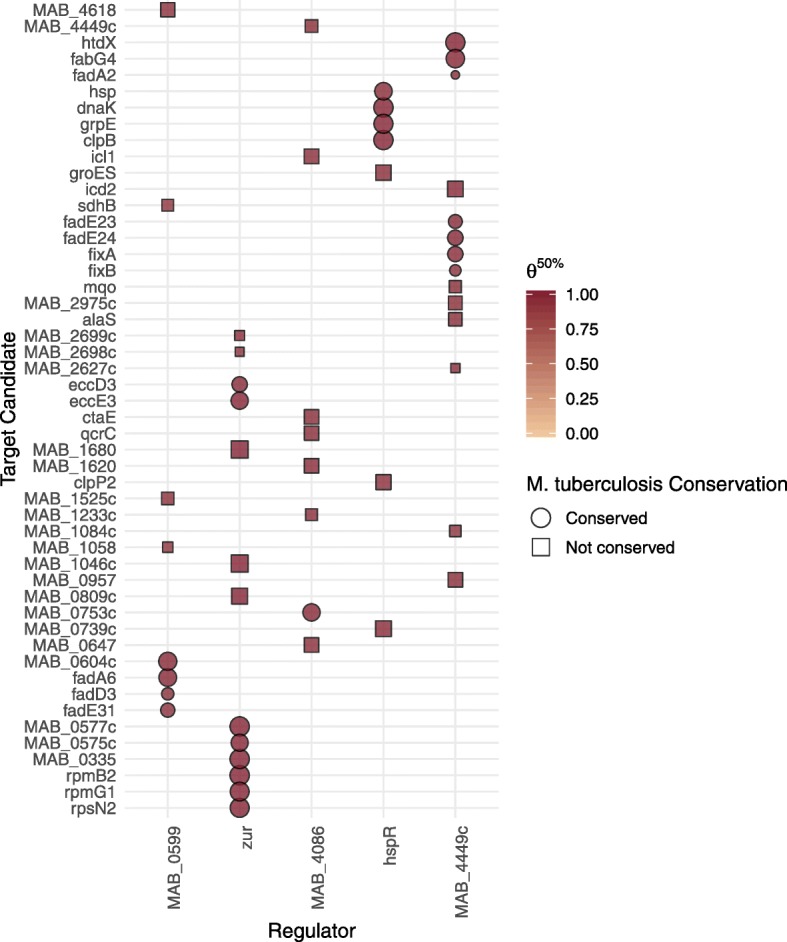
Abacus plot illustrating interaction candidates achieving $\theta _{r,t}^{50\%} > 0.9$ for the uninformative parameterisation; larger points are suggestive of less uncertainty; circles correspond to validated regulatory interactions in *M. tuberculosis*; shading corresponds to the posterior $\theta _{r,t}^{50\%}$ estimate. Regulators and targets are arranged by genomic position

Of the *N*=167,280 regulator-target pairs considered in *M. abscessus*, under the uninformative parameterisation, BINDER identified 54 pairs across 5 transcription factors with a posterior 50th percentile $\theta _{r,t}^{50\%} > 0.9$ (Table 3). Of these 54 interactions, 24 are known to have validated orthologous regulatory interactions in *M. tuberculosis* as per ChIP-seq data (Fig. 7); the number of interaction pairs almost doubles by reducing the threshold by 0.1 (102 pairs with 31 known orthologous interactions satisfying $\theta _{r,t}^{50\%} > 0.8$). In comparison, under the informative parameterisation, a similar effect was observed with 54 pairs with 21 known orthologous interactions satisfying $\theta _{r,t}^{50\%} > 0.9$. A more conservative effect was observed for the precise settings: 33 pairs across 28 transcription factors with a posterior 50th percentile $\theta _{r,t}^{50\%} > 0.9$. As expected, for all parameterisations, the vast majority of posterior distributions of ***θ*** were centred at low values, suggesting low levels of regulatory connectivity across the *M. abscessus* interactome; the mean 50th percentile for all of ***θ*** was 0.085 (sd: 0.106) for the uninformative parameterisation and 0.087 (sd: 0.105) and 0.0885 (sd: 0.0995) for the informative and precise parameterisations respectively. It should be noted that in the benchmarking exercise (“Application of BINDER to *Escherichia coli* and *Bacillus subtilis* data” section) we observed that the nominal value of a regulator-target pair’s $\theta _{r,t}^{50\%}$ is not always as informative as its relative magnitude to {*θ*_*r*,1_,…,*θ*_*r, N*_}. In general, whilst there were many instances of plausible conserved interactions, the results suggest evidence for many non-conserved interactions that may be unique to *M. abscessus*. Further, it can be observed that for a given regulator, many of the regulated genes appear to be spatially clustered along the genome (Fig. 7). This observation lends support to the concept of gene colocalization arising as a means to affect efficient transcription [42, 43].

**Table 3:** Regulator-target pairs achieving a posterior $\theta _{r,t}^{50\%} > 0.9$ in *M. abscessus* by regulator under the uninformative parameterisation

Regulator Locus Tag	Regulator Gene Name	Total Interactions	Conserved Interactions	Unconserved Interactions
MAB_0599	-	8	4	4
MAB_1678c	zur	15	8	7
MAB_4086	-	8	1	7
MAB_4270c	hspR	7	4	3
MAB_4449c	-	16	7	9

The parameter *ζ*_*r*_ in the auxiliary component influences the inferred probability of regulator-target interaction before any further regulator-target pair information is taken into account, with larger values of *ζ*_*r*_ meaning higher interaction probabilities. In this sense, each *ζ*_*r*_ is related to the ubiquity of regulation by regulator *r* across the genome. Under the uninformative parameterisation, we observed an average posterior mean of -6.63 across all regulator models (sd: 4.07). Hence, intuitively, conditional on the auxiliary data ME and PE being zero, the probability of a regulatory interaction is low.

**Fig. 8:**
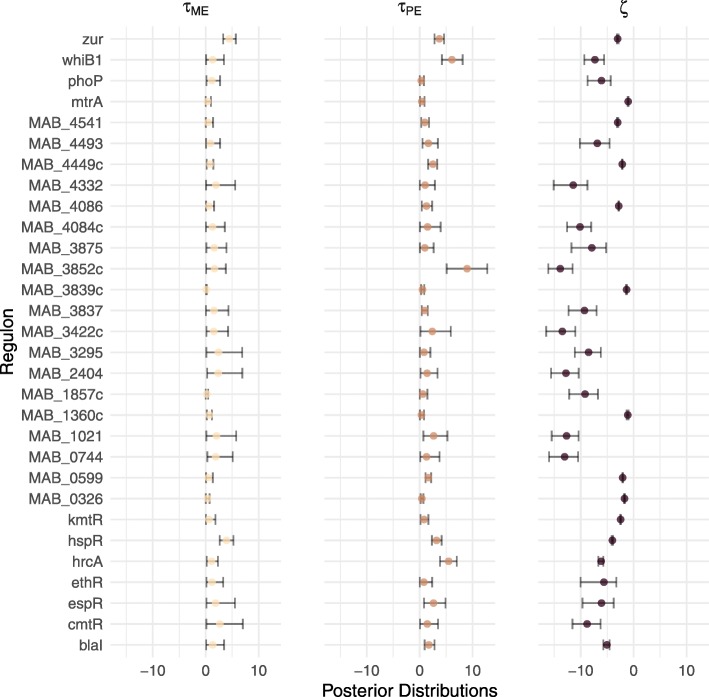
Central 95% of mass of the posterior distributions for $\protect \phantom {\dot {i}\!}\tau _{\text {ME}_{r}}$, $\protect \phantom {\dot {i}\!}\tau _{\text {PE}_{r}}$ and *ζ*_*r*_ under the uninformative parameterisation with posterior means indicated by dots for each of the *R*=34 regulators

The parameter $\phantom {\dot {i}\!}\tau _{\text {ME}_{r}}$ captures the influence the auxiliary ME data has on the prior mean of the inferred probability of a regulatory interaction between regulator *r* and target *t*, given all other covariates. Across all regulators, under the uninformative parameterisation, we observed an average posterior mean for $\phantom {\dot {i}\!}\tau _{\text {ME}_{r}}$ of 1.43 (sd: 0.9982) (Fig. 8). The parameter $\phantom {\dot {i}\!}\tau _{\text {PE}_{r}}$ has a similar interpretation for the auxiliary data PE. Across all regulators, under the uninformative parameterisation, we observed an average posterior mean for $\phantom {\dot {i}\!}\tau _{\text {PE}_{r}}$ of 1.95 (sd: 1.8981) (Fig. 8). These results suggest that, on average, both ME and PE are positively correlated with the primary data in the likelihood. Given the phenomenon of genomic conservation, this is as we would expect and lends credence to the BINDER approach. Furthermore, although the mean posterior means for $\phantom {\dot {i}\!}\tau _{\text {ME}_{r}}$ and $\phantom {\dot {i}\!}\tau _{\text {PE}_{r}}$ are quite similar, the latter has larger variation suggesting higher volatility in the influence of PE than in the influence of ME.

**Fig. 9:**
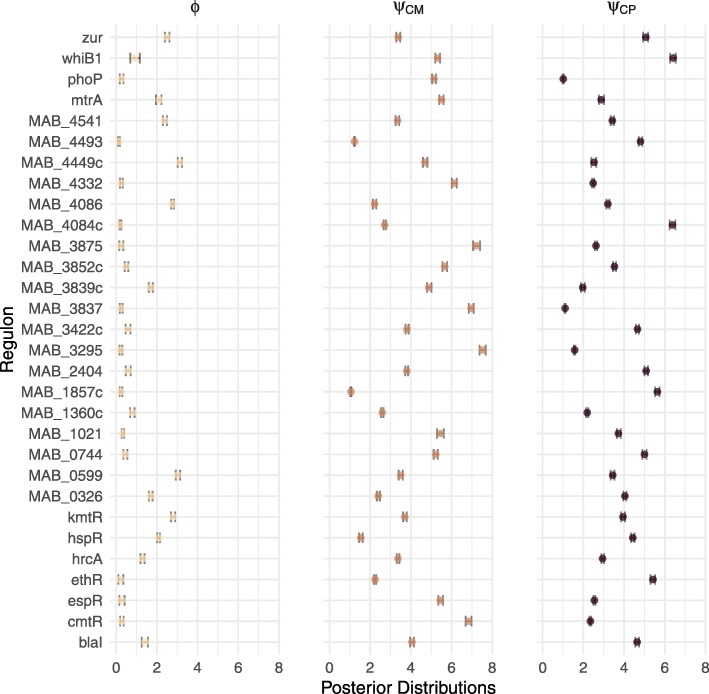
Central 95% of mass of posterior distributions for *ϕ*_*r*_, $\protect \phantom {\dot {i}\!}\psi _{\text {CM}_{r}}$ and $\protect \phantom {\dot {i}\!}\psi _{\text {CP}_{r}}$ under the uninformative parameterisation with posterior mean values denoted by dots for each of the *R*=34 regulators

In terms of scale parameters, under the uninformative parameterisation, ***ϕ*** tended to have the lowest posterior mean values (average posterior mean of 1.12 with standard deviation 1.0067) (Fig. 9). Both $\phantom {\dot {i}\!}\psi _{\text {CM}_{r}}$ and $\phantom {\dot {i}\!}\psi _{\text {CP}_{r}}$ yielded larger posterior mean estimates. In particular, under the uninformative parameterisation, $\psi _{\text {CM}_{r}}\phantom {\dot {i}\!}$ yielded an average posterior mean of 4.23 (sd: 1.7713) and $\phantom {\dot {i}\!}\psi _{\text {CP}_{r}}$ yielded an average posterior mean of 3.63 (sd: 1.4499), suggesting that the primary CM data tend to lie further from logit(*θ*_*r, t*_) than CP (Fig. 9). Also, the larger average posterior mean associated with $\phantom {\dot {i}\!}\psi _{\text {CM}_{r}}$ compared with that of $\phantom {\dot {i}\!}\psi _{\text {CP}_{r}}$ is intuitive, given the extra uncertainty associated with motif inference (comprised within CM) compared with validated orthologous interactions comprised within CP.

#### Interpretation of results: composition of the zur regulon

As an example of a putative discovery facilitated by BINDER, we examine the inferred regulon corresponding to the transcriptional regulator zur (MAB_1678c). The zur regulator present in *M. tuberculosis* and *M. abscessus* is a zinc-responsive transcription factor. Zinc is an essential element for life in many organisms [44]. In addition to its role as a structural scaffold for many proteins, it fulfils a critical function as a frequent enzyme and DNA-binding protein cofactor [45]. However, zinc can be toxic at high concentrations [46]. For prokaryotes, efficient zinc acquisition, concentration and tolerance are critical processes for survival and pathogenicity [47]. Zinc homeostasis in prokaryotes is achieved via cellular import and export, zinc binding, and zinc-sensing [47]. Cellular zinc levels are maintained by importer and exporter proteins which are then regulated at the transcriptional level by several zinc-responsive transcription factors [48], including the zur regulator.

As per ChIP-seq results, the original regulon pertaining to zur in *M. tuberculosis* (Rv2359/furB) comprised 26 target genes (12 directly regulated targets); under the uninformative parameterisation, of these targets, 14 (53.8%) contained orthologs in *M. abscessus*. Using the cutoff criterion $\phantom {\dot {i}\!}\theta _{\text {zur},t}^{50\%} > 0.9$, BINDER suggested 15 target candidate genes in *M. abscessus* be considered valid targets of zur, 8 of which correspond to evidenced interactions in *M. tuberculosis*. Gene ontological analysis carried out on the putative targets provided intuitive insight, revealing up-regulated biological processes (*p*≤0.05) corresponding to metal ion transport.

BINDER also identified a number of interesting non-conserved putative targets for zur. For example, MAB_1046c, is annotated as a cobalamin synthesis protein. This is interesting as MAB_0335, one of the identified conserved targets, is also annotated as a cobalamin synthesis protein. This is perhaps owing to the role of cobalamin as a cofactor for cobalamin dependent methionine synthase in prokaryotes. Cobalamin dependent methionine synthase is involved in zinc ion binding [49]. Further, MAB_2698c and its immediately adjacent neighbour MAB_2699c also yield high $\theta _{\text {zur},t}^{50\%}$ posterior estimates; gene ontology suggests that MAB_2699c, another unconserved putative target, is involved in pseudouridine synthesis/pseudouridine synthase activity; pseudouridine synthases catalyse the isomerisation of uridine to pseudouridine in RNA molecules and are thought to act as RNA chaperones. Intriguingly, pseudouridine synthase I (TruA) [50], one of the four distinct families of pseudouridine synthases, contains one atom of zinc essential for its native conformation and tRNA recognition [51]. Another unconserved target is the PPE-like gene MAB_0809c; PPE genes are widely considered to play a key role in pathogenesis. Interestingly, phagosomes containing PPE genes found to disrupt lysosome-phagosome fusion have been shown to display differences in zinc levels relative to corresponding phagosomes containing PPE-knockout mutants [52]. Another highly-probable unconserved interaction, MAB_1680, is annotated as a putative transmembrane protein. Given its association with zur, MAB_1680 is perhaps involved with zinc uptake in *M. abscessus*.

## Discussion

In this work we have inferred the GRN in *M. abscessus* using the BINDER approach, the primary purpose of which is to infer the probability of pairwise interactions in a collection of regulator-target pairs. BINDER exploits experimental coexpression data in tandem with the property of genomic conservation to probabilistically infer a GRN in *M. abscessus*. To infer a GRN, BINDER proceeds by binding information from data in primary and auxiliary strata.

BINDER facilitates information sharing horizontally (by sharing parameters in the same layer of the model hierarchy) and vertically (by sharing of parameters in distinct strata of the hierarchy). The likelihood function assumes independence of the assumed logit-normal distributed primary data variables, conditional on the shared parameter of interest *θ*_*r, t*_, representing the probability of an interaction in the (*r, t*)^*th*^ regulator-target pair. Further, the mean of this interaction probability’s logit-normal distribution is informed by a linear function of the auxiliary data, serving as a proxy for genomic conservation information. Thus inference is strengthened through the borrowing of information across variables and strata.

With the exception of PE, the construction of all variables considered (i.e. ME, CM and CP) involves the choice of thresholds and/or decisions. For example, from the outset we have formed a TFBS-based module binary membership structure and an orthologous target binary membership structure, recorded in the auxiliary binary variables ME and PE respectively, on which the primary variables CM and CP rely. However, in order to circumvent potential loss of information associated with such hard membership, a “soft" approach using scale free topology or clustering coefficients may be worth exploring. Under these scenarios, the idea of membership has a continuous representation [15]. Further, the auxiliary variable ME is derived from thresholding a *p*-value and as such is sensitive to the cutoff point *ε* selected. The BINDER approach also implements a further two threshold points *δ*_CM_ and *δ*_CP_; clearly it is of paramount importance to choose these thresholds in an informed and careful manner. We have employed a hypergeometric framework for CM and CP, but any mapping to [ 0,1] is possible. Again, topological overlap mapping or clustering coefficent mapping [15] are alternative approaches. With a view to foregoing the need to choose a threshold at all, simply mapping a regulator-target pair to the mean of its coexpression with members of the ME and PE modules is possible because the mean of a group of unsigned coexpressions will also lie in [0,1]; validation studies suggests that this approach, although convenient, does not perform quite as well as the hypergeometric framework.

It should be noted that, for our purposes, we had a relatively small-scale expression compendium with which to form our coexpression networks. Both the volume and diversity of RNA-seq conditions used to construct the coexpression networks may not be fully sufficient to computationally infer the entire GRN in *M. abscessus*. Small coexpression data sets are more likely to comprise noisy correlation results and similar experimental conditions have the effect of duplicating expression information leading to low numbers in terms of effective sample sizes. Similarly, for some regulators, we observed a lack of specificity in binding sites (owing to very long binding regions and small numbers of binding interactions); this has the effect of negatively impacting motif inference (i.e. false discovery of erroneous motifs). Naturally, more reliable data are preferable, however where data are less reliable, it is possible to account for this uncertainty through specification of the hyperparameters in the priors on the variable-specific parameters. Regardless, as the signal deteriorates (e.g. erroneous consensus motifs, inaccurate binding interactions), inference will suffer and thus it is important to ensure that all data sources are as accurate as possible. For the above reasons, it may be worthwhile to examine the more conservative BINDER parameterisations (i.e. the precise parameterisations) detailed above. This parameterisation implements a less diffuse prior distribution such that candidates lacking auxiliary support are less likely to achieve high *θ*_*r, t*_ estimates.

Through the course of this analysis, with a view to focusing on inferred highly probable regulator-target interactions, we have examined pairs for which the posterior median $\theta ^{50\%}_{r,t} > 0.9$. However, the intention behind this model is not to define interaction probability on the basis of a single point estimate, but rather to provide a posterior distribution of *θ*_*r, t*_. This allows for a more nuanced analysis on interaction probability estimates than is typically provided by a simple binary classifier. Instead, we recommend that estimates are received in the context of the scientific question posed; varying the the number and severity of thresholds and tolerances will allow for differing results. Similarly, as noted in the fur regulon inference for *E. coli* explored in the benchmarking results, under certain scenarios BINDER estimates low values for all interaction candidates (both positive and negative cases); this is either due to influential hyperparameter settings and/or poor agreement between the auxiliary and primary data. However, even under these scenarios, BINDER can still estimate higher estimates for positive interaction cases. In such cases, as is good statistical practise, prior sensitivity analyses should be conducted or it may be worthwhile to consider regulator results individually.

One obvious limitation of any model that exploits conservation phenomena to perform inference in scarcely annotated organisms is that such a model can only make inference based on existing conservation data; indeed BINDER cannot infer interaction that may exist in *M. abscessus* on regulators not considered here. There are modelling approaches for “de novo" network inference that are based exclusively on coexpression analysis or other non-conservation based predictors, but such approaches can contain many false positives [53]. Instead BINDER aims to overcome such issues by allowing coexpression-based data have partial influence on model inference. Moreover, while BINDER requires a consensus sequence motif and a collection of orthologous regulator-target interactions to perform inference, it is possible to run BINDER with a consensus sequence motif *or* a collection of orthologous interactions only. In this case, BINDER comprises one variable in the auxiliary stratum and one variable in the primary stratum.

One mechanism used by cells to refine and maintain transcription factor levels is autoregulation. It has been argued that the occurrence of autoregulation positively correlates with the developmental or physiological importance of the transcription factor [54]. Given that any gene will have a perfect coexpression with itself, most expression-based approaches (such as GENIE3 and iRafNet) to GRN inference are unable to detect transcription factor autoregulation. For a given regulator, BINDER uses the coexpression profiles of a target gene with genes under the control of the regulator to inform the probability of a regulator-target interaction. BINDER does not examine the coexpression of the target candidate with regulator directly. As a result, BINDER is able to detect autoregulation.

For each regulator considered here, we applied the BINDER approach to all 4920 annotated protein-coding genes in *M. abscessus*. However, in theory, BINDER could be applied to any desired subset of genes. With a view to accurately describing whole-population behaviour we recommend including all available data, albeit acknowledging the associated additional computational cost.

Pearson’s correlation was employed here as a measure of coexpression. Although there are other options, with a view to remaining conservative and reducing false positives, Pearson’s correlation gives high values when expression values are strongly linearly related. Common alternatives include the more flexible Spearman’s method, but often with increased flexibility comes an increase in less biologically significant relationships. Although use of Pearson’s correlation can come at the cost of increased false negatives, studies have suggested that many coexpression relationships are linear and monotonic so this issue may be overstated [55].

Recent studies have suggested that implementing an ensemble approach to motif identification can improve detection results [56]. BINDER could be extended to augment the number of motif search tools used in the analysis. Similarly, another suggestion might be to augment the number of proxy organisms from a single proxy organism to *k* proxy organisms, similar in vein to [24]. A spike-and-slab prior distribution [57] for the associated model parameters would provide insight on the information contained in the individual proxy organisms. Furthermore, it is possible to extend the dimensionality of the primary stratum. In general, data that are binary or lie in [ 0,1] can be appended to the primary stratum: for example, the direct coexpression between a given regulator-target pair could be used to form a trivariate primary stratum. Although we have used exclusively binary variables in the auxiliary stratum, there is no restriction on the form of auxiliary data that can be modelled by BINDER.

It may be worthwhile to investigate the effect of incorporating more sophisticated levels of dependency in the BINDER model. Such dependencies could be based on operon comembership, on regulator family membership (e.g. the whiB-like family [58]), on target reoccurrence or on gene function using GO [59] or COG [60], for example. Here, we only consider the gene immediately downstream of a confirmed or putative TFBS to be under the regulation of the associated regulator. Recent studies suggest that operon organisation is dynamic and, hence, operon structures are capable of changing across conditions [61]. However, given that BINDER considers not only the existence of a precedent interaction and/or motif match for a given candidate, but also the coexpression of that candidate with other candidates that do comprise a precedent interaction and/or motif match, BINDER is capable of detecting adjacent gene coregulation. Members of operon structures that are cotranscribed across all conditions considered will exhibit greater coexpression than those that are only cotranscribed under a fraction of conditions considered; as a result, BINDER is able to reflect that behaviour through the *θ*_*r, t*_ posteriors. Furthermore, it is possible to construct prior distribution parameterisations such that BINDER will tend to estimate higher *θ*_*r, t*_ median values for genes in cotranscribed structures if they comprise a precedent interaction and/or motif match; this may facilitate the determination of gene importance in cotranscribed structures. Owing to the lack of assumptions made by BINDER with respect to transcription start sites and operon co-membership, we expect that the results generated by BINDER will sufficiently aid in the generation of dynamic regulatory networks, as well as the understanding of transcriptional unit plasticity.

## Conclusions

We have sought to determine the evidence for gene regulation in *M. abscessus* using a range of expression data from *M. abscessus* and experimentally validated regulatory network data from *M. tuberculosis*. We have demonstrated the extent to which there is a correlation between gene regulation in *M. tuberculosis* and transcriptome coexpression in *M. abscessus*. Our results imply not only strong genic conservation between *M. abscessus* and *M. tuberculosis* but also evidence of conservation with respect to the modes of transcriptomic control between these two organisms.

We have implemented a Bayesian modelling approach to quantifying the probability of an interaction across a collection of 167,280 regulatory-target pairs. Of these, 54 regulator-target pairs, across 5 transcription factors, were inferred to have a posterior 50th percentile for *θ*_*r, t*_>0.9 in *M. abscessus*.

The interactions identified in this study will form a valuable resource for further studies of transcriptional control in *M. abscessus* and in the family of *Mycobacteriaceae* more generally. Further, the BINDER framework is applicable across a wider range of organisms for which similar data are available.

## Methods

### Data

Given the paucity of data available from the primary organism *M. abscessus* (MAB), BINDER integrates data from a proxy organism *M. tuberculosis* (MTB) into the inferential procedure. Specifically, we leverage data from across orthologous ChIP-seq validated interactions in *M. tuberculosis* as proxy data and extract the primary data from 32 RNA-seq libraries across 16 distinct experimental conditions in *M. abscessus*. Thus we consider the set of all possible regulator-target interaction candidate pairs, arising from the set *R*=34 orthologous regulators in *M. tuberculosis*, and *T*=4920 target genes in the *M. abscessus* genome yielding *N*=167,280 regulator-target pairs of interest.

#### Auxiliary data: motif evidence (ME) and precedent evidence (PE)

**Motif Evidence:** With respect to a given regulator *r*, the TFBS status of a target *t* is encoded through a binary variable termed motif evidence (ME). Specifically, for a regulator-target pair, ME takes the value 1 if the corresponding target contains a putative TFBS for the regulator’s motif in its upstream region and a value of 0 otherwise. Here, the binding motif is assumed to be identical to the binding motif in the proxy organism.

With a view to determining regulator motifs, we extracted binding sequences using the NCBI *M. tuberculosis* (Accession: AL123456) complete chromosome sequence and annotation, *S*_MTB_. The evidenced binding region coordinates were provided by ChIP-seq data sets ranging across several induced transcription factor experiments in *M. tuberculosis*. We subsequently categorised these binding sequences by regulator with a view to discovering binding sequence consensus motifs. The MEME motif discovery tool [62] was used to infer a single consensus binding motif *M*_*r*_ for each regulator *r*∈*R*: in particular, using a DNA alphabet, we searched on both strands seeking zero or one occurrence per binding sequence of a single consensus motif between 10 and 30 nucleotides long.

To find putative TFBSs for the derived motifs in the *M. abscessus* genome, we defined a sequence region *U*_*t*_ corresponding to the region -300nt to +50nt of the start of each target of interest *t*∈*T*. This interval size was chosen in light of the distribution of intergenic region lengths in the *M. abscessus* genome. In order to find putative TFBSs for each *M*_*r*_, we searched in each *U*_*t*_ using the complete chromosome sequence and annotation *S*_MAB_ provided by NCBI for *M. abscessus* (Accession: NC010397). In the scenario that the most upstream coordinate of an immediately adjacent upstream gene was annotated to occur within 300nt of an upstream region of interest, the upstream region of interest was truncated to the most upstream coordinate of the upstream gene. To perform this search, we used the FIMO tool [63] to find the high-scoring upstream sequences with a *q*-value ≤*ε*=0.1. We provided a background file encoding 0-order nucleobase probabilities based on all upstream sequences of interest.

In summary, for each regulator-target pair (*r, t*) for *r*=1,…,*R* and *t*=1,…,*T* the motif evidence ME_*r, t*_ is computed where:

$$ \text{ME}_{r,t} = \left\{\begin{array}{ll} 1 & \text{if for \(M_{r}\) the FIMO {q}-value for}\ U_{t} \leq \epsilon \\ 0 & \text{otherwise.} \end{array}\right. $$

For a given regulator *r*, we refer to the set of all genes where ME_*r, t*_=1 as the ‘ ME_*r*_ module’.

**Precedent Evidence:** The presence of an annotated orthologous regulator-target interaction in the proxy organism is encoded in the binary variable termed precedent evidence (PE). For a regulator-target pair, PE takes the value of 1 if such an orthologous interaction exists and takes the value of 0 otherwise.

Specifically, given both the proxy genome *G*_MTB_ and the primary genome of interest *G*_MAB_, Ortholuge [64] derived one-to-one orthologs were used to map orthologous regulator-target interactions from *G*_MTB_ to *G*_MAB_. ChIP-seq data sets drawn from 34 induced transcription factor experiments in *G*_MTB_ were scanned for orthologous regulator-target interactions with respect to *G*_MAB_; orthologous regulator-target pairs were subsequently grouped by regulator to derive a rudimentary orthology of regulons in *G*_MAB_.

Thus, given the rudimentary orthology, for a given regulator *r* and target *t*:

$$ \text{PE}_{r,t}\! =\! \left\{\!\begin{array}{ll} 1 & \text{if orthologous evidence of}\ r \text{ regulating}\ t \text{ in}\ G_{\text{MTB}} \\ 0 & \text{otherwise.} \end{array}\right. $$

As in the ME case, for a given regulator *r*, we refer to the set of all genes where PE_*r, t*_=1 as the ‘ PE_*r*_ module’.

#### Primary data: coexpression of motif and precedent evidence

**Coexpression of Motif Evidence:** Exploiting the property that genes sharing a common regulator exhibit strong coexpression [14], we computed a measure termed coexpression of motif evidence (CM). For a given regulator, using the motif derived from the proxy organism, CM quantifies the extent to which a target gene coexpresses with genes that have strong affinity for the putative regulator motif in the primary organism.

Specifically, for a regulator binding sequence motif *M*_*r*_ inferred from *G*_MTB_, we define CM_*r, t*_ for a given gene regulator-target pair (*r, t*) in *G*_MAB_. We define the reduced primary genome $\phantom {\dot {i}\!}G_{\text {MAB},-O_{t}} = G_{\text {MAB}} \setminus O_{t}$, where *O*_*t*_ is a *t*-inclusive set of genes in *G*_MAB_ that should not be used in the calculation of CM_*r, t*_. This set will naturally include *t*, but can contain any other genes that are not desired for calculation of CM_*r, t*_. The variable CM_*r, t*_ lies in [ 0,1], where values closer to 1 represent stronger correlation between expression levels of the target *t* with genes in $\phantom {\dot {i}\!}G_{\text {MAB},-O_{t}}$ producing strong matches to the inferred sequence motif *M*_*r*_. Specifically, for a regulator-target pair

$$ \text{CM}_{r,t} = \left\{\begin{array}{ll} \text{hypergeometric}(a|b, c, d) & \text{ for}\ a,b,d \geq 1\\ 0 & \text{ otherwise} \end{array}\right. $$

where hypergeometric(*a*|*b, c*,*d*) represents the cumulative distribution function of a hypergeometric random variable *a* with parameters *b*, *c* and *d* where, for some threshold *δ*_CM_,

- *a* is the number of genes in $\phantom {\dot {i}\!}G_{\text {MAB},-O_{t}}$ that belong to the ME_*r*_ module and have an absolute expression correlation with gene *t* >*δ*_CM_
- *b* is the number of genes in $\phantom {\dot {i}\!}G_{\text {MAB},-O_{t}}$ exhibiting an absolute expression correlation with gene *t*>*δ*_CM_
- *c* is the number of genes in $\phantom {\dot {i}\!}G_{\text {MAB},-O_{t}}$ exhibiting an absolute expression correlation with gene *t*≤*δ*_CM_
- *d* is the number of genes in $\phantom {\dot {i}\!}G_{\text {MAB},-O_{t}}$ that belong to the ME_*r*_ module.

A Benjamini and Hochberg adjustment [65] is applied to these probabilities to relax the observed polarisation of probabilities around 0 and 1; for a given regulator *r*, the adjustment is relative to all targets *t*∈*T*. We expect genes under the control of regulator *r* to coexpress strongly with members of the ME_*r*_ module. For our purposes, we vary the threshold such that each *δ*_CM_ is specific to each target. For a given target *t*, assuming CX_*i, j*_ represents the coexpression between genes *i* and *j*, we choose *δ*_CM_ to be equal to the 95th percentile of all values in the set $\phantom {\dot {i}\!}\{\text {CX}_{t,g} \text { for}\ g \in G_{\text {MAB},-O_{t}}\}$.

**Coexpression of Precedent Evidence:** Analogous to CM, we develop a score of coexpression of precedent evidence, CP. For a given regulator, CP quantifies the extent to which a target gene coexpresses with orthologs of genes comprising regulator-target interactions in the proxy organism.

Specifically, for regulator *r*, we define the regulon *P*_*r*_ as the collection of orthologous interactions annotated in *G*_MTB_. For a given gene regulator-target pair (*r, t*) in *G*_MAB_ the variable CP_*r, t*_ is defined on the interval [ 0,1], where values closer to 1 represent stronger expression correlation of gene *t* with orthologs of genes from *P*_*r*_ in $\phantom {\dot {i}\!}G_{\text {MAB},-O_{t}}$. That is,

$$ \text{CP}_{r,t} = \left\{\begin{array}{ll} \text{hypergeometric}(a | b, c, d) & \text{ for}\ a, b, d \geq 1\\ 0 & \text{ otherwise} \end{array}\right. $$

where, for a threshold *δ*_CP_

- *a* is the number of genes in $\phantom {\dot {i}\!}G_{\text {MAB},-O_{t}}$ that belong to the PE_*r*_ module and have an absolute expression correlation with gene *t*>*δ*_CP_
- *b* is the number of genes in $\phantom {\dot {i}\!}G_{\text {MAB},-O_{t}}$ containing an ortholog in *G*_MTB_ and exhibit an absolute expression correlation with gene *t*>*δ*_CP_
- *c* is the number of genes in $G_{\text {MAB},-O_{t}}\phantom {\dot {i}\!}$ containing an ortholog in *G*_MTB_ and exhibit an absolute expression correlation with gene *t*≤*δ*_CP_
- *d* is the number of genes in $G_{\text {MAB},-O_{t}}\phantom {\dot {i}\!}$ that belong to the PE_*r*_ module.

Again, the probabilities are subject to Benjamini and Hochberg adjustment relative to all target candidates *t*∈*T*. We expect genes under the control of regulator *r* to coexpress strongly with members of the PE_*r*_ module. Thus again we choose *δ*_CP_ to be equal to the 95th percentile of all values in the set $\phantom {\dot {i}\!}\{\text {CX}_{t,g} \text { for}\ g \in G_{\text {MAB},-O_{t}}\}$.

With a view to quantifying coexpression in *G*_MAB_, the expression profiles (using RPKM [66]) of all genes constituting the NCBI GenBank annotation for the *G*_MAB_ genome were computed across 32 RNA-seq libraries (comprising 16 distinct experimental conditions) elicited from a range of astringent response and control experiments. In order to compute the corresponding coexpression profiles, we generated the unsigned Pearson correlation coefficient of all possible pairwise annotated gene-pair combinations. All read files were aligned using Bowtie (version 1.2.2) [67] and totalled using Samtools (version 1.7) [68]. RNA-seq libraries can be found on NCBI’s Gene Expression Omnibus (Accession: GSE78787).

### The BINDER model for inferring a GRN

Borrowing strength across the primary and auxiliary data sets, we computationally infer the GRN for *M. abscessus* through a novel statistical modelling approach: BayesIan gene regulatory Networks inferreD via gene coExpression and compaRative genomics (BINDER). BINDER is a Bayesian hierarchical model that appositely models the type and structure of both the primary and auxiliary data to infer the probability of a regulatory interaction between a regulator-target pair candidate. Each of *N*=|*R*|×|*T*| observations is a regulator and target candidate pair (*r, t*) from the set of regulators *R* and the set of target candidates *T* in the *M. abscessus* genome. Interest lies in the probability *θ*_*r, t*_ of there being an interaction between regulator *r* and target *t*. Thus, inferring *θ*_*r, t*_ facilitates inference of the *M. abscessus* GRN.

As stated, BINDER integrates primary data from *M. abscessus* with data from the proxy organism *M. tuberculosis*. Specifically, the variables CM and CP (“Primary data: coexpression of motif and precedent evidence” section) constitute the primary data stratum whilst ME and PE (“Auxiliary data: motif evidence (ME) and precedent evidence (PE)” section) constitute the auxiliary stratum. As BINDER is a Bayesian hierarchical model, the auxiliary data inform the prior distribution for each *θ*_*r, t*_; the posterior distribution for each *θ*_*r, t*_ is then updated by accounting for the primary data.

To define the likelihood function of the BINDER model we appositely model the primary data type and assume logit-normal distributions for CM and CP. As such, in the case where CM_*r, t*_ or CP_*r, t*_ were 0 or 1, they were increased or decreased respectively by a small factor (10^−4^). Further we assume, given *θ*_*r, t*_, the regulator-target pairs and primary variables are conditionally independent:

$$ {\begin{aligned} \mathcal{L}&(\boldsymbol{\theta}, \psi_{\text{CM}}, \psi_{\text{CP}} | \boldsymbol{\text{CM}}, \boldsymbol{\text{CP}})\\ & = \prod_{\substack{ r \in R \\ t \in T}} \mathcal{N}_{l}\{\text{CM}_{r,t} | \text{logit}(\theta_{r,t}),\psi_{\text{CM}_{r}}\} \mathcal{N}_{l}\{\text{CP}_{r,t} | \text{logit}(\theta_{r,t}),\psi_{\text{CP}_{r}}\} \end{aligned}}  $$

Here $\mathcal {N}_{l}(x | a, b)$ denotes the logit-normal distribution of *x* with location and standard deviation parameters *a* and *b* respectively. The location parameter is common across the distributions for CM and CP. This shared parameter enables the borrowing of information across variables, in addition to facilitating tractability through the conditional independence assumption. The conditional independence assumption is widely employed in other settings, such as latent class analysis [69, 70].

As with any Bayesian hierarchical model, prior distributions are specified on the BINDER model parameters. For each *θ*_*r, t*_ we posit a logistic normal prior such that $\text {logit}(\theta _{r,t}) \sim \mathcal {N}(\gamma _{r,t}, \phi)$ where *ϕ* is the standard deviation parameter controlling the level of dispersion around the mean. The mean *γ*_*r, t*_ is informed by the auxiliary data ME and PE on the regulator-target pair (*r, t*) through a linear model. Specifically:

1 $$\begin{array}{@{}rcl@{}} \gamma_{r,t} & = & \zeta_{r} + \tau_{\text{ME}_{r}} \text{ME}_{r,t} + \tau_{\text{PE}_{r}} \text{PE}_{r,t}  \end{array} $$

Independent priors are then posited on the parameters in (1) such that the intercept $\zeta _{r} \sim \mathcal {N}(\mu _{\zeta },\sigma _{\zeta })$ and a truncated normal prior is assumed on the slope parameters: $\tau _{k_{r}} \sim \mathcal {N}_{(0,\infty)}(\mu _{\tau _{k}},\sigma _{\tau _{k}}) \text { for}\ k \in \{\text {ME},\text {PE}\}$. This truncated normal prior with mass on the positive real line reflects the assumption that the presence of regulation in regulator-target pair (*r, t*) in the proxy organism is suggestive of the presence of such regulation in *M. abscessus*. To complete the model setup, prior distributions are placed on the scale parameters such that $\psi _{l_{r}} \sim \mathcal {N}_{(0,\infty)}(\mu _{\psi _{l}},\sigma _{\psi _{l}}) \text { for}\ l \in \{\text {CP},\text {CM}\}$. The hyperparameters of all the specified prior distributions must be set by the practitioner and their values are potentially influential; sensitivity of inference to their choice is explored in “Prior sensitivity analysis” section.

In order to infer the GRN for *M. abscessus*, the set of parameters {*θ*_*r, t*_:*r*∈*R, t*∈*T*} are of primary interest. Thus the required posterior distribution is

$$ {\begin{aligned} p&(\boldsymbol{\theta} | \boldsymbol{\text{CM}}, \boldsymbol{\text{CP}}, \boldsymbol{\text{ME}}, \boldsymbol{\text{PE}}, \boldsymbol{\mu}, \boldsymbol{\sigma})\\ & = \int_{\boldsymbol{\tau}} \ldots \int_{\boldsymbol{\psi}} p(\boldsymbol{\theta}, \boldsymbol{\psi}, \boldsymbol{\phi}, \boldsymbol{\tau}, \boldsymbol{\zeta} | \boldsymbol{\text{CM}}, \boldsymbol{\text{CP}}, \boldsymbol{\text{ME}}, \boldsymbol{\text{PE}}, \boldsymbol{\mu}, \boldsymbol{\sigma}) d\boldsymbol{\psi} d\boldsymbol{\phi} d\boldsymbol{\zeta} d\boldsymbol{\tau} \end{aligned}}  $$

**Fig. 10:**
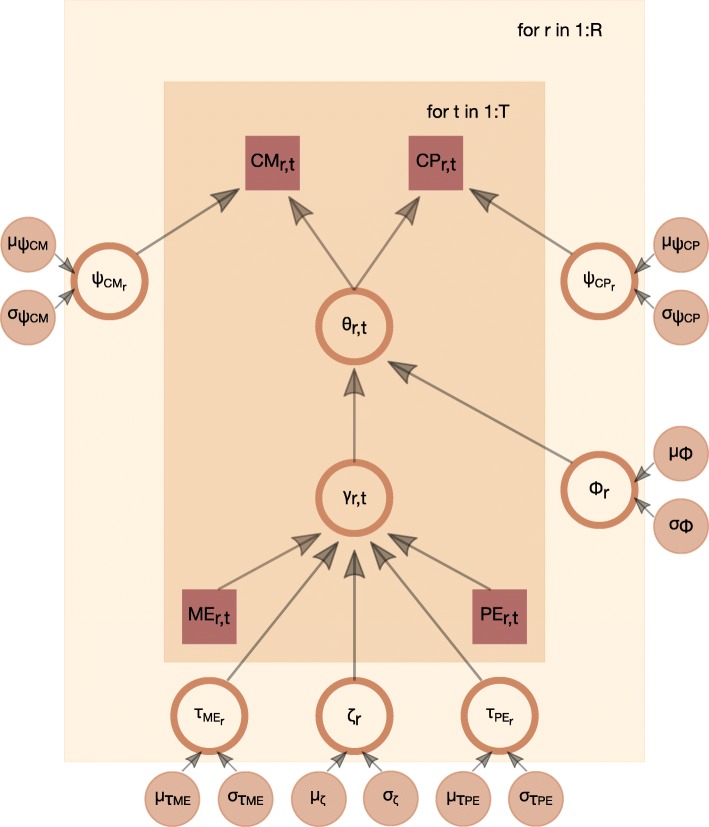
Graphical representation of the hierarchical BINDER model; squares correspond to observed data, large discs correspond to random parameters and small discs correspond to fixed hyperparameters; the surrounding boxes denote observation-specific parameters and data

This posterior distribution is explored using Stan [71], a state-of-the-art platform for statistical modelling and computation for large data sets that employs Hamiltonian Monte Carlo methods [72] to draw samples from the posterior distribution of interest. An illustration of the BINDER model is provided in Fig. 10.

## Data Availability

An implementation of the BINDER approach is available as an R package at github.com/ptrcksn/BINDER. The datasets generated and analysed in the current study are available at github.com/ptrcksn/BINDER_paper_analysis .

## Abbreviations

AUC: Area under curve
*B. subtilis*: *Bacillus subtilis*
BINDER: BayesIan gene regulatory networks inferreD via gene coExpression and compaRative genomics
ChIP-Seq: Chromatin immunoprecipitation followed by sequencing
CM: Coexpression of motif evidence
CP: Coexpression of precedent evidence
DNA: Deoxyribonucleic acid
*E. coli*: *Escherichia coli*
GRN: Gene regulatory network
*L. monocytogenes*: *Listeria monocytogenes*
*M. abscessus*: *Mycobacterium abscessus*
*M. tuberculosis*: *Mycobacterium tuberculosis*
MAB: *Mycobacterium abscessus*
MAD: Mean absolute deviation
ME: Motif evidence
MTB: *Mycobacterium tuberculosis*
NCBI: National center for biotechnology information
PE: Precedent evidence
PPE: Proline-proline-glutamate
RPKM: Reads per kilobase per million
*P. aeruginosa*: *Pseudomonas aeruginosa*
RBB: Reciprocal-best-BLAST
RNA: Ribonucleic acid
RNA-seq: RNA sequencing
ROC: Receiver operating characteristic
SSD: Supporting-species-divergence
TFBS: Transcription factor binding site
tRNA: Transfer ribonucleic acid
